# Progress of Surgery

**Published:** 1904-01-23

**Authors:** 


### PROGRESS OF SURGERY.

### GENITO-URINARY.

Cystoscopy and Cryoscopy.—Kümmell (Hamburg),[1] at the meeting of the German Surgical Association in June, once more discussed the newer methods of examination in cases of renal disease and their influence on the success of renal operations. The methods alluded to comprised cryoscopy of the blood and of the urine from each kidney, *i.e.*, the determination of the freezing point of these fluids, the use of the *x*-rays and of the catheterizing cystoscope, and the determinating of the electrical resistance of the urines from each kidney. He has determined the freezing point of the blood in 500 cases, and in all the cases obtained useful and reliable results. By the methods of research alluded to it is possible, without exploratory cystotomy or laparotomy, to determine the presence of a second kidney, and whether or not the second kidney, if present, is capable of accomplishing the work of the other kidney if that one be removed. With the help of the above methods the author has succeeded in performing 72 nephrectomies without a death. Discussing the use of the Roentgen rays for the detection of renal calculus, the author states that he has gained the conviction that any stone in the kidney is visible on a Roentgen plate if the proper technical measures for the production of these photographs have been taken; also he believes that the absence of a shadow equally certainly shows the absence of a stone. This is true, however stout the patient and whatever the size or composition of the stone. It is necessary to take many photographs, and in all of them the shadow of the stone must be constant in position and contour. Usually the shadow is a few centimetres from the spine and slightly below the twelfth rib. Skilled examination of the plates is necessary and good illumination. The author examines the plates in a dark room, placing them before lights of varying intensity contained in a case, and he examines them with an opera-glass. The objections which have been raised to catheterization of the ureters appear to the author to rest on theoretical rather than on practical grounds, and he believes that no one who has mastered the technique of the procedure can be found to oppose its use. The results yielded by the various urine segregators and separators, which he has tested, have not been found as reliable as those given by catheterization, but these instruments are to be recommended where catheterization is impossible. It is very rarely necessary to give a general anæsthetic in order to catheterize the ureters; it is sufficient to inject a 1 or 2 per cent. eucaine solution or to wash out the bladder with a 1 per cent. antipyrin solution. For the estimation of the functional capacity of the kidneys the author has tried various methods, including the use of methylene blue, the phloridzin test, the determination of the density of the urine by measuring its electrical resistance, and cryoscopy, and has found the last, viz, cryoscopy of the blood and urine, to be the simplest and the most accurate. The principle which underlies this method is that the urine and the blood in the kidneys obey the laws of osmosis. Korányi found that in men there was great constancy of the osmotic concentration of the blood if the kidneys were normal, but an increase of the concentration in renal disease and with the latter a diminution of the concentration of the urine. The osmotic concentration of the fluids in question is determined by finding their freezing-points, for the more concentrated a solution the lower is its freezing-point below that of distilled water. By concentration in this connection is meant the "molecular concentration in the osmotic sense" which has to be distinguished from specific gravity. The newest mode of testing the urine from each kidney, by determining the electrical resistance, rests upon the same principles, and the results of this method and of cryoscopy have been found to run parallel. The electrical method can be employed with smaller quantities of urine, and takes less time than cryoscopy. To get reliable results from cryoscopy requires considerable care and practice, for errors of technique and observation easily creep in. The following are some of the author's conclusions:—(1) With intact kidneys the molecular concentration of the blood is constant and corresponds to a freezing point of −0·56°C. or to between −0·57C. and −0·55C. (2) With bilateral kidney disease usually there is an increase of the concentration of the blood and a diminution of that of the urine, and if this is not present then one of the kidneys is capable of doing the work of both, even if it is not quite healthy. (3) One-sided kidney disease does not cause increase of the blood concentration. (4) Nephrectomy is not to be recommended if the freezing point of the blood is depressed to −0·6°C. (5) With even slight disease, either of the secreting substance or of the pelvis of one kidney, the other kidney being normal, cryoscopy will show depression of the freezing point of the urine from the diseased as compared with that from the healthy side. The author's paper contains many other interesting observations which we have not space to abstract.

[1] Beilage zum Zentralb. f. Chir., Sept. 5, 1903, p. 110.

*(To be continued.)*

